# The Monocrotaline Model of Hypertension Leads to Cachexia in Male but Not Female Mice

**DOI:** 10.1002/jcsm.70129

**Published:** 2025-11-20

**Authors:** Abbey L. Politeski, Alexander Q.L Rico, Jack Cambell, Julio G. Cisneros Medrano, Ben Witmer, Risha Gupta, Noah A. Fiorucci, Katherine M. Lycett, Hannah C. Smith, Hannah M. Kavanagh, Cayleih E. Robertson, Ingrid K. M. Brenner, Aaron B. A. Shafer, Holly E. Bates, Kirk Hillsley, Stephanie W. Tobin

**Affiliations:** ^1^ Department of Biology Trent University Peterborough Ontario Canada; ^2^ Environmental and Life Sciences Graduate Program Trent University Peterborough Ontario Canada

**Keywords:** cardiac cachexia, hypertension, lipolysis, monocrotaline, muscle atrophy

## Abstract

**Background:**

The monocrotaline (MCT) model of cardiac cachexia is a pharmaceutical approach to pulmonary hypertension that has been used to study heart failure and muscle wasting in rodents; however, little is known of how this pyrrolizidine alkaloid leads to peripheral changes in organ function and body mass, and sex differences have not been adequately compared.

**Methods:**

Ten‐ to 12‐week‐old male and female C57BL/6N mice were treated weekly with MCT (200 mg/kg) for 8 weeks. Body weight, feeding behaviour and stool output were monitored weekly. In the final week, endurance was measured via a treadmill fatigue study. Upon termination, organs were weighed and processed for histochemistry and downstream gene expression analysis.

**Results:**

Males were more susceptible to MCT‐induced weight loss. No change in gross heart or skeletal muscle mass was observed in either sex, though lung mass was elevated in both sexes and cardiomyocyte size was larger in males (*p* < 0.05). MCT reduced the cross‐sectional area of the tibialis anterior muscle in both sexes (*p* < 0.05), but this did not correspond to changes in endurance, as the treadmill fatigue study revealed no change in total time or distance run in response to MCT in either sex. RNA‐seq analysis of the gastrocnemius muscle showed no significant changes in gene expression when compared within either the male or female cohort (*n* = 3), but when pooled (*n* = 6), MCT reduced gene pathways associated with mitochondrial function, adipogenesis and DNA repair and upregulated pathways associated with inflammation. Total fat mass was reduced by 40% in male mice in response to MCT, mainly because of significant reductions in inguinal white and brown interscapular adipose tissue mass. This was independent of food intake and intestinal distress, as no differences in stool wet:dry output or feeding behaviours were observed in either sex. Gene expression and immunohistochemical analysis of inguinal fat suggest that adipose tissue within males is particularly sensitive to MCT, as *Tnfa*, *Ppargc1a* and *Zfp516* were upregulated, and there was a significant interaction between sex and MCT on *Retn* and *Pnpl2a* (aka Atgl) expression.

**Conclusions:**

These data suggest sex‐dependent physiological responses of mice to MCT, where adipose tissue loss is more pronounced in males as a result of adipose tissue inflammation and metabolic activation. In contrast, conserved atrophic effects of MCT are observed within skeletal muscle, irrespective of sex. Further research using this model should consider sex‐dependent responses.

## 1 Introduction

Cachexia is a complex multiorgan syndrome characterized by an imbalance between protein synthesis and degradation, inflammation and, most importantly, the involuntary loss of skeletal muscle and fat tissue [1]. In cases of metabolic stress or inflammation, the rates of protein degradation increase disproportionally in comparison with the rate of protein synthesis, causing significant muscle loss and worsening of disease prognosis [2]. Cachexia is a consequence of advanced chronic conditions such as cancer, HIV/AIDS and heart failure [1, 3, 4]. The condition is associated with late‐stage disease, high mortality rates and an overall decline in the quality of life [5]. In the context of heart failure, this condition is referred to as cardiac cachexia, and it is estimated to affect between 5% and 15% of heart failure patients [1]. Given the higher incidence of cachexia in cancer patients (50%–80%), most research on cachexia has been done in preclinical cancer models, and the development of a reliable model of cardiac cachexia has been a major limitation in understanding this condition. The monocrotaline (MCT) model of cardiac cachexia is a pharmaceutical approach to pulmonary hypertension, often used to study heart failure and muscle wasting in rodent models [1, 6]. MCT is a pyrrolizidine alkaloid derived from the plant *Crotalaria spectabilis* and is metabolized into its active form, dehydromonocrotaline (aka monocrotaline pyrrole; MCTP), by cytochrome P‐450 (CYP3A4) [7]. MCT metabolites function as both hepatotoxins and pneumotoxins, which circulate through the bloodstream after being produced in the liver and eventually reach pulmonary blood vessels, where they inflict damage on the capillary endothelium [7, 8, 9, 10, 11]. In this model, pulmonary hypertension is attributed to endothelial damage, which leads to vascular hyperresponsiveness and the thickening of the medial vascular smooth muscle layer in the pulmonary blood vessels; this leads to right ventricular hypertrophy and eventually to heart failure [8, 10]. The development of pulmonary hypertension by MCT is also accompanied by persistent inflammation: Studies using rats have shown that an acute inflammatory phase develops within 6 days after treatment in which vascular remodelling occurs; this is followed by a chronic inflammatory phase in which vascular remodelling worsens [11].

MCT has also been used to model cardiac cachexia in rats and mice; however, as mice are somewhat resistant to MCT‐induced pulmonary hypertension, ongoing MCT administration at a significantly higher dosage is required to observe similar physiological responses. This has been suggested to be due to differences in the metabolism of MCT to MCTP [12]. In mice, these weekly treatments have ranged from 80 to 600 mg/kg of body weight [13, 14, 15, 16, 17], whereas rats develop pulmonary hypertension with just a single dose of 60 mg/kg [12]. There are also potential differences in how male and female rodents respond to MCT, as male rats have increased P‐450 3A4 activity [18]. Most studies that used MCT focused on only one sex, typically male rats and mice. A limited number of studies have demonstrated a cardioprotective effect in female rats in response to MCT, but none have extended these findings to measure impacts on cachexia and muscle loss [19, 20]. Notably, the cardioprotection in female rats is lost after ovariectomy [21], and female foetuses are more sensitive to MCT exposure in utero [22], which suggests that differences in P‐450 3A4 activity may not be responsible for a dampened response to MCT. To our knowledge, no such investigation into the potential sexual dimorphic responses of cardiac and skeletal muscle to MCT has been completed in mice.

The ambiguity of the MCT model, particularly in mice, raises questions about the extent to which findings can be generalized to humans for a comprehensive understanding of cardiac cachexia. Given the reported differences in sensitivity and the potential influence of biological sex on MCT metabolism and/or tissue and organ resiliency, a detailed evaluation of the effects of MCT on multiple organs is warranted. Therefore, the aim of this study was to evaluate the effects of MCT on the progressive changes in body mass, feeding behaviour, stool output and endurance in addition to measurements of terminal organ weights. RNA‐seq was used to evaluate widespread changes in skeletal muscle biology. Furthermore, as the potential for sex‐dependent responses to MCT has been proposed in other contexts [19, 23], we used both male and female mice to evaluate these outcomes. We found that compared with males, female mice are generally protected from the effects of MCT in terms of changes in total body weight and organ mass and adipose tissue inflammation. These data demonstrate sex‐dependent responses in this model, which should be considered in future studies.

## 2 Methods

### 2.1 Animal and Study Design

Animal procedures were approved by the Trent University Animal Care Committee. All animals were subject to similar conditions, housed at room temperature and subject to a 12:12 light–dark cycle. Unless otherwise indicated, standard chow and water were provided ad libitum. A total of 28 male and 29 female C57BL/6N (Charles River) mice aged 10–12 weeks were used in this study. To induce hypertension and cardiac cachexia, mice were dosed weekly with 200 mg/kg of MCT (Cayman Chemical Company, 315‐22‐0) via subcutaneous injection. Briefly, MCT was suspended to a stock concentration of 50 mg/mL in dimethyl sulfoxide (DMSO) and further diluted in saline, resulting in an injection volume of approximately 8.8% DMSO. Half of each sex (i.e., 15–16) were treated with MCT, whereas the other received a control injection of saline and DMSO. Mice were further subdivided, and half of each group was used for food intake and stool measurements and thus singly housed (*n* = 7–8 per group). Each animal received a total of eight injections over 8 weeks and was terminated 1 week after the final injection. ‘Week 1’ corresponds to the first injection, whereas ‘Week 9’ corresponds to the date of termination. Control animals for each sex received an equivalent volume of saline and DMSO. Body weight, food consumption and stool output were recorded weekly. Mice were sacrificed following anaesthetization with isoflurane and cervical dislocation. Wet weights of muscles (gastrocnemius and tibialis anterior), heart, fat pads (gonadal, inguinal and the interscapular brown adipose pads), lungs (including dry weight) and liver were recorded and normalized to tibia length. Intestinal length was also recorded.

### 2.2 Measurement of Exercise Capacity

Exercise capacity was determined in Week 8 using a treadmill fatigue test as previously described [24]. Mice underwent a 2‐day training protocol to acclimate them to the treadmill, during which a wire brush was used to gently poke mice at the rear of the treadmill to encourage running. On the first day of training, the speed was set to 8 m/min and gradually increased to 10 m/min over a 10‐min period. On the second day of training, the speed was set to 10 m/min and gradually increased to 12 m/min over a 15‐min period. After training, mice were allowed to rest for 1 day with no treadmill exposure between the training and the fatigue test. The fatigue protocol began at 12 m/min, increasing by 2 m/min over 75 min, at which time the test was ended. The fatigue test was performed with the shock grids off. Mice were removed from the treadmill when fatigue was detected or when 75 min had elapsed. Criteria for fatigue‐like behaviour included remaining at the rear of the treadmill, on the shock grid, or one body length away from the shock grid, for 5 s continuously. Distance (m) = Time spent running (min) × speed (m/min).

### 2.3 Food Intake Measurements

Mice were singly housed for these experiments. Ad libitum food consumption was recorded weekly. At Week 7, a fasting refeeding study was conducted wherein mice were fasted for 4 h prior to monitoring a 24‐h ad libitum feeding window. To measure ad libitum feeding within a 24‐h window after fasting (Week 7) or in a nonfasted state (Week 8), food weight was measured at the onset of the dark cycle, again 2 h later (i.e., a 2 h window between 7 and 9 pm), at the start of their light phase the next day (i.e., a 10‐h window between 9 and 7 am) and finally at the end of the 24‐h cycle at the onset of the next dark cycle (representing a 12 h window between 7 am and 7 pm). The 2‐h window was included to measure the acute drive to eat after the 4‐h fast but was also performed in the 24‐h ad libitum.

### 2.4 Stool Measurements

Like the feeding studies, mice were singly housed. Intestinal activity was analysed throughout the study via measurement of stool dry weight rate within a 24‐h window. The stool was dried for 72 h at 50°C after the wet weight was recorded. The water content of stool was calculated as a percentage by the difference between wet and dry stool weight, divided by wet stool weight.

### 2.5 Cardiac Histology

After tissues were weighed, hearts were fixed in 10% formalin for 48 h, then rinsed with water and moved to 70% ethanol. Tissues were embedded in paraffin, sectioned at 6 μm on positively charged slides and stained with haematoxylin and eosin (H&E). Cross‐sectional area (CSA) was calculated using ImageJ.

### 2.6 Immunofluorescence

At sacrifice, tibialis anterior muscles were immediately fixed in 4% paraformaldehyde at 4°C for 24 h. Samples were placed in a 15% and then 30% sucrose gradient overnight at 4°C, in succession. Samples were embedded in optimal cutting temperature (OCT) compound (23‐730‐571, Fisher Scientific). Seven‐micrometre sections were obtained using a cryostat, mounted on positively charged slides. Samples were blocked in 5% normal goat serum (NGS; NEB #5425S) and 0.3% Triton X‐100 (NEB #39487S). An antilaminin rabbit monoclonal antibody (Sigma‐Aldrich L9393) was prepared (1:1000) in antibody dilution buffer (1× PBS, 1% bovine serum albumin [NEB #9998S]). Antirabbit goat IgG conjugated to Alexa Fluor 555 (NEB #4413) was then added (1:1000). Finally, the slides were stained with Prolong Gold Antifade Reagent with DAPI (NEB #8961) and allowed to cure overnight at room temperature. Tissues were examined under a microscope and imaged, and the CSA of 150 fibre areas per animal was taken using ImageJ.

### 2.7 RNA‐Seq

To examine differential gene expression in the muscle tissue of the mice, RNA sequencing was performed on 12 of the mice (*n* = 3 per group). The gastrocnemius muscles used in RNA‐sequencing were immediately submerged in RNAlater and later processed using TRI reagent according to the manufacturer's instructions (AM9738, Thermo Fisher Scientific), followed by processing with an RNA cleanup kit (T2040 or T2050, Monarch). The mRNA libraries (NEBNext) were sequenced at 2 × 150 bp, 20–30 M reads per sample on a Novaseq X 10B flow cell (The Centre for Applied Genomics, Toronto, Canada). Quality control metrics were assessed using Fastqc v0.12.1 on the raw reads. The sequencing reads were then trimmed using Trimmomatic and the following threshold values: (1) trailing = 10, (2) leading = 10, (3) base quality threshold = 10. We mapped the reads against the mouse reference genome GCRm38–mm10 with the minimum alignment score function L,0,‐0.2 using hisat2 v2.2.1. Read quantification was performed with Featurecounts using a minimum overlap value of 1 and differentially expressed genes were calculated using normalized read counts and an adjusted *p*‐value < 0.05 and 0.05 < log_2_FC < −0.5 (DESeq2). Gene set enrichment analysis (GSEA) was performed in R (version 4.5.0) using the fgsea package for pathway enrichment and msigdbr for curated gene sets. Gene expression data were first ranked by log_2_ fold change values using precomputed results from differential expression analysis. The gene sets used were obtained from the MSigDB Hallmark collection via msigdbr (species = ‘ *Mus musculus* ’, collection = ‘H’). The GSEA was conducted using the fgseaMultilevel function. Significantly enriched pathways were defined as those with adjusted *p*‐value (FDR) < 0.05. To visualize the enrichment, plotEnrichment from the fgsea package was used to generate individual plots for each pathway. The top 10 significantly upregulated pathways (based on the highest normalized enrichment score, NES > 0) and the top significantly downregulated pathways (NES < 0) were selected and visualized as multipanel figures using the cowplot package.

### 2.8 Adipose and Cardiac Tissue Homogenization, cDNA Conversion and qPCR Analysis

Hearts were collected, weighed and either placed into RNALater (Sigma‐Aldrich) or flash frozen in liquid nitrogen. The tissues were then transferred into a prechilled mortar and pulverized under liquid nitrogen using a pestle until they were a fine powder. All tools and tissues were chilled frequently to prevent the tissue from melting. The pulverized heart was transferred to a cool microcentrifuge tube, weighed and stored at −80°C until RNA isolation. RNA was isolated using RNAzol (Sigma‐Aldrich, St. Louis, MO, USA, #R4533). Inguinal adipose tissue was pulverized into a fine powder over liquid nitrogen. RNA was subsequently extracted using the RNeasy Lipid Tissue Mini Kit (cat. no. 74804) from QIAGEN according to the manufacturer's instructions. Cardiac and adipose tissue RNA was converted to cDNA using the LunaScript RT SuperMix Kit protocol from New England Biolabs (NEB #E3010). Relative gene expression was assessed using the Luna Universal qPCR Master Mix Protocol (NEB M3003) and analysed using the delta delta Ct method. Primers are listed in Table S1.

### 2.9 Adipose Tissue Processing and H&E Staining

Brown and white adipose tissues were placed into plastic embedding cassettes and fixed in 4% paraformaldehyde at 4°C for 24 h. The tissue samples were then placed into a tissue processor (Thermo‐Fisher Scientific Shandon Excelsior ES Histology) and run through serial ethanol solutions (70% for 45 min, 90% for 90 min, 95% for 75 min and 100% for 60 min each), followed by xylene (60 min each), wax (58°C paraffin wax for 4 h each), and then stored at 4°C until embedding. After processing, samples were embedded into paraffin wax blocks (Sakura Finetek Tissue‐Tek TEC 5). Adipose tissue sections were prepared using a microtome (Thermo‐Fisher Scientific Shandon Finesse ME) at a thickness of 5 μm and fixed onto charged microscope slides (Leica Biosystems X‐tra adhesive microscope slides, 3800200). Ten to 15 slides with two to three sections on each were taken for each tissue sample. H&E staining: Slides were placed into two xylene solutions for 10 min each to deparaffinize sections. After deparaffinization, samples were rehydrated using a graded series of ethanol solutions of decreasing concentration in the following order: 100% (2×) for 10 min each, 90% for 5 min and 70% for 5 min. After rehydration, the tissue was rinsed with deionized water for 1 min followed by running tap water for 5 min to prepare it for haematoxylin or UCP1 staining. For H&E, the slides were placed into haematoxylin (Sigma‐Aldrich Mayer's Haematoxylin Solution, MHS16) for 15 min followed by a tap water rinse for 10 min and then dipped into an ammonia (0.025%) water solution for 30 s. This was followed by a brief tap water rinse for 15 s before staining with eosin (Leica Biosystems Eosin Y Powder, Product Number: 3803805). The slides were placed into 5% aqueous eosin for 2 min and then dehydrated with increasing concentrations of ethanol solutions in the following order: 70% (1×) for 15 s, 90% (1×) for 15 s and 100% (2×) for 2 min each. The slides were then washed with xylene, using two successive solutions for 2 min each, and then cover‐slipped with Permount (Electron Microscopy Sciences Permount Mounting Medium, 5027798) and allowed to dry before further analysis. Staining was done using a Sakura Finetek Tissue‐Tek Prisma Slide stainer.

### 2.10 Adipose Tissue Immunohistochemistry

Slides were prepared as described above. Heat‐mediated antigen retrieval with Universal HIER antigen retrieval buffer (Abcam, AB208572) was performed for 20 min in a rice cooker, cooled with running tap water for 10 min and rinsed with PBS. The slides were then washed twice with phosphate‐buffered saline (PBS) (10 min each). Hydrogen peroxide blocking reagent (Abcam, AB64218) was applied for 10 min at room temperature, followed by two 10‐min rinses in PBS with tween 20 (PBS‐T). Samples were blocked in 10% goat serum (Abcam, Ab7481) in a humidity chamber for 1 h at room temperature. This step was followed by a PBS‐T wash for 10 min with shaking. Samples were incubated with anti‐UCP1 (Abcam, Ab209483) at a 1:4000 dilution with blocking buffer, overnight in a humidity chamber at 4°C. Prior to incubation with the secondary antibody, a second round of endogenous peroxidase blocking was performed. Samples were then incubated with goat antirabbit secondary antibody conjugated to horse radish peroxidase (HRP) at a 1:500 dilution with blocking buffer in a humidity chamber at room temperature for 1 h and then washed three times in PBS‐T for 15 min each. 3,3′‐Diaminobenzidine (DAB) solution (Abcam, Ab64328) was applied for 10 min and then washed with running tap water until the solution was clear and counterstained with Harris's Haematoxylin (Sigma‐Aldrich, HHS32) for 2 min and rinsed with tap water until the solution runs clear. The slides were then dehydrated with increasing concentrations of ethanol solutions (70% for 3 min, 90% for 3 min and 100% for 3 min) and fixed with xylene (5 min). Finally, slides were mounted with Permount (Fisher Chemical, SP15‐100) mounting medium and allowed to dry overnight before further analysis.

### 2.11 Quantification of Multilocular Area, UCP1^+^ Cells and Cell Density Within Brown and White Adipose Tissue

After staining, slides were randomly assigned a number and subsequently analysed by a blinded individual using the Leica DM500. Ten to 12 images were taken per slide, evenly distributed to ensure accurate representation of the entire tissue section, with no overlap. The images for each tissue section were then analysed using imaging software (ImageJ). For each image, the total adipose tissue area was first measured in mm^2^ by outlining the area with a freehand tool. After the total adipose tissue area was measured, the unilocular and multilocular areas were also measured separately using the freehand tool to determine the percentage of multilocular area within each image and tissue section. To measure UCP1 levels, 10 images were taken per slide, evenly distributed following a zig‐zag line across the section to ensure accurate representation of the entire tissue section, with no overlap. After the total adipose tissue area was measured, positive UCP1 was measured using the colour deconvolution plugin for haematoxylin and DAB (H DAB) staining and selecting the DAB (brown) image. The UCP1 positive area was then measured by setting a threshold to select the coloured area of the image. The percentage of UCP1‐positive area was then calculated using the total tissue area of the image. Cell density within BAT was calculated using the haematoxylin (blue image) from the colour deconvolution plugin (H DAB). The total number of nuclei stained by the haematoxylin was then counted using the multipointer tool to determine the total cell count of the tissue area (including all cells, not adipocytes only). The cell density was then calculated by dividing the total cell count for each image by the total adipose tissue area for each image to determine the number of cells per mm^2^.

### 2.12 Statistical Analyses

Data were analysed using Prism 10. Data were analysed via two‐way ANOVA and Šidák's test for multiple comparisons. Only male control vs. male MCT and female control vs. female MCT were compared. A Student's *t*‐test was used to compare cardiomyocyte and skeletal myofiber CSA. A three‐way ANOVA was used to identify differences in feeding behaviours for Figure 4. A *p*‐value of < 0.05 was considered statistically significant. Exact ANOVA *p*‐values for interaction and main effects are shown in Table S2.

## 3 Results

### 3.1 MCT Induces Weight Loss in Male but Not Female Mice and Is Associated With Sex‐Dependent Cardiac Hypertrophy

**FIGURE 1:**
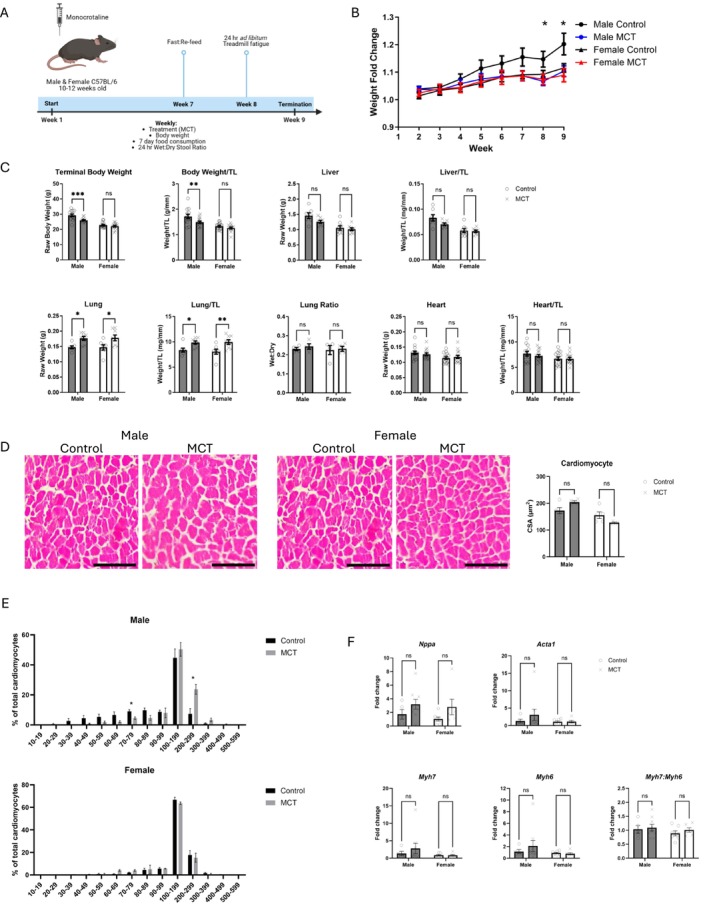
Multiorgan and cardiovascular dysfunction in male and female mice in response to monocrotaline. (A) An overview of the experimental procedures using weekly injections of monocrotaline (MCT). Male and female C57BL/6N mice, aged 10–12 weeks, received weekly injections of MCT (200 mg/kg) for 8 weeks and were terminated at Week 9, 1 week after the 8th injection. This image was made in Biorender. (B) Weekly changes in body mass of animal groups as calculated relative to Week 1 (*n* = 13–15). (C) Terminal body and organ mass shown as raw weight or normalized to tibia length (TL). Data are shown as mean ± SEM. (D) Cardiomyocyte cross‐sectional area (CSA) and representative images of each group stained with haematoxylin and eosin (H&E). Scale bar = 300 μm, *n* = 4–5. (E) Binned analysis of cardiomyocyte CSA represented as a percentage of total cardiomyocytes (*n* = 4–5). (F) Cardiac gene expression, presented as fold change relative to *Ubiquitin C* (*Ubc*), *n* = 5–9*.* For all analyses except Panel E, a two‐way ANOVA with Šídák's post hoc analysis was used to calculate statistical significance. *p < 0.05, **p < 0.01, ***p < 0.001, ***p < 0.001. For Panel E, a *t*‐test was used to determine statistical significance (*p < 0.05).

Mice received 200 mg/kg of MCT (weekly for 8 weeks) via subcutaneous injection (Figure 1A). The effects of MCT on body mass in males manifested in Weeks 8 and 9, as a significant decrease in body mass was observed in response to MCT; however, this effect was not seen in females (Figure 1B–C; interaction effect of terminal body weight *p* = 0.025). Weekly raw body measurements are found in Table S3. We measured liver mass as MCT is also known to induce hepatotoxicity [25], but there was no significant change in liver mass in either sex. Studies using higher concentrations of MCT (e.g., 600 mg/kg) increase lung and heart weight in male mice [13, 16]. In our study using 200 mg/kg, lung mass (normalized to tibia length) was heavier in both sexes, but there was no change in heart mass or the lung wet:dry ratio in either sex (Figure 1C; main effect of MCT on lung mass *p =* 0.0005). Normalized terminal liver and heart mass showed main effects for sex (*p* < 0.05).

To further probe for cellular and molecular markers of cardiac hypertrophy, cardiomyocyte CSA and genes associated with hypertrophy were measured. We observed an interaction between sex and treatment in cardiomyocyte CSA, though no significant difference with post hoc analysis (Figure 1D–E; interaction *p* = 0.0090). Binned measurements of individual cardiomyocyte size show cardiomyocytes are larger in males but not females in response to MCT, as indicated by a right‐shifted curve and a significant increase in cardiomyocytes 200–299 μm in size. We next probed for expression of hypertrophy‐related genes, *Nppa* (coding for atrial natriuretic peptide), *Myh6*, *Myh7* and *Acta1* (Figure 1F). There was no significant change in expression of these genes, though a main effect was observed for *Nppa* in response to MCT (*p* = 0.0469).

### 3.2 MCT Reduces Skeletal Muscle Myofiber Size in Both Sexes

**FIGURE 2:**
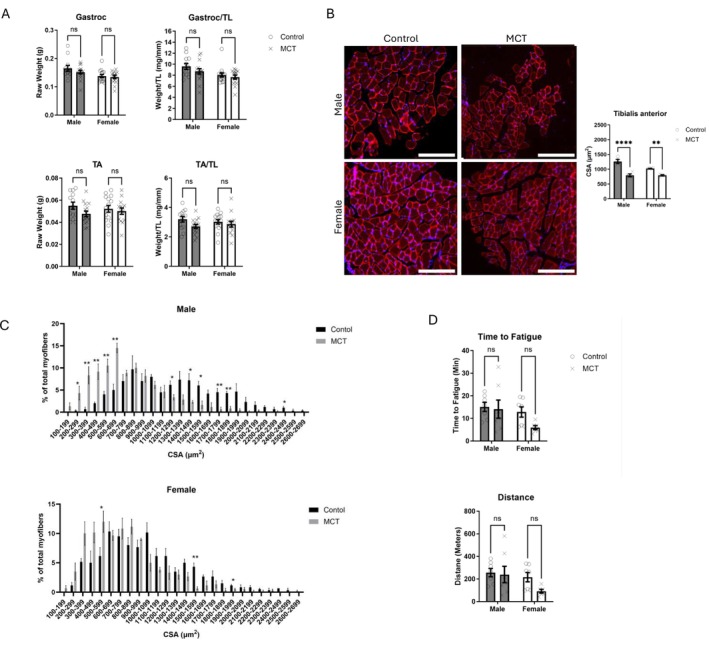
Physiological responses of skeletal muscle to monocrotaline. (A) Terminal muscle mass of the tibialis anterior (TA) and gastrocnemius (Gastroc) muscles. Both raw mass and mass normalized to tibia length (TL) are shown (*n* = 13–15). (B) Cross‐sectional area (CSA) of the tibialis anterior muscle (*n* = 4). The bottom panels depict representative immunofluorescent images from each group for Laminin (red) and Dapi (blue). Scale bar = 200 μm. (C) Frequency plots of myofiber size, presented as the percentage of total myofibers (*n* = 4). Top, males; Bottom, females. (D) Endurance testing (distance, time to fatigue, work and power). Animals were subject to a treadmill fatigue test in Week 8 (*n* = 6–7). Data are shown as mean ± SEM. A two‐way ANOVA with Šídák's post hoc was used to calculate statistical significance. **p < 0.0, ****p < 0.0001. For Panel C, a *t*‐test was used to determine statistical significance (*p < 0.05, **p < 0.01).

Skeletal muscle mass and function were assessed next. The mass of hindlimb muscles, the tibialis anterior (TA) or gastrocnemius (gastroc), was not significantly affected by MCT, though there was a main effect of sex on gastroc weight (Figure 2A; *p* < 0.01). The CSA of the TA was significantly reduced in both male and female MCT‐treated mice (Figure 2B; interaction *p* = 0.0197). When myofiber CSA was quantified in a frequency plot, both male and female MCT‐treated mice demonstrated a left‐shifted curve, characteristic of muscle atrophy (Figure 2C). A treadmill fatigue study showed no change in distance or time to fatigue, though female MCT‐treated mice tended to perform worse than their control counterparts in both experiments (Figure 2D). There was no significant main effect of sex or treatment on the treadmill exercises. No change in intramuscular fat was detected via Oil Red O staining (data not shown).

### 3.3 Transcriptional Analysis Reveals a Downregulation in Mitochondrial and Muscle‐Related Genes in Response to MCT

**FIGURE 3:**
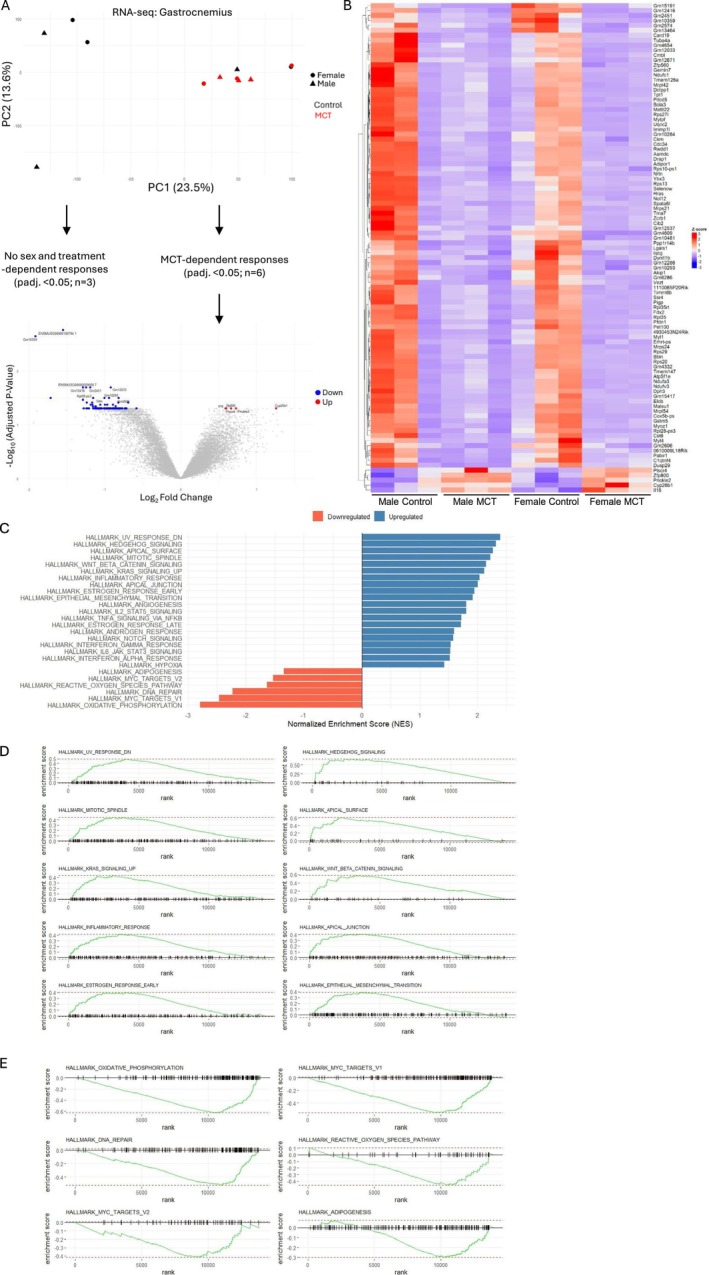
RNA‐seq analysis of the gastrocnemius muscle. (A) Principal component analysis demonstrates clustering of MCT‐treated samples, independent of sex, and that two control samples had strong overlap with the MCT‐treated group. Subsequent analysis of differentially expressed genes (DEGs) pooled control and MCT samples, independent of sex, leading to 118 DEGs, as outlined in the volcano plot. Three female controls and three male control samples were pooled into one control group (*n* = 6), and a similar approach was taken for the MCT group (*n* = 6). (B) A heatmap depicts the total number of differentially expressed genes (0.05 < log_2_FC < −0.5, *p*adj. < 0.05). (C) Normalized enrichment scores of pathways identified by gene set enrichment analysis (GSEA). Significantly enriched pathways were defined as those with adjusted *p*‐value (FDR) < 0.05. (D) Enrichment plots of the top 10 (out of 20) upregulated pathways. (E) Enrichment plots of the top 6 (out of 6) downregulated pathways.

Despite no apparent changes in muscle mass in response to MCT, we hypothesized that the change in CSA could reflect biological changes in gene expression. Therefore, RNA‐seq of the gastrocnemius muscle was conducted to better understand how MCT may be affecting muscle biology. Principal component analysis (PCA) showed a general clustering of MCT‐treated mice (red circles and triangles) compared with the control group (black circles and triangles), though there was variability in the control samples (Figure 3A). Each sex was first assessed individually (i.e., male control vs. male MCT; female control vs. female MCT). No difference in gene expression was detected within the female cohort, and only two genes were differentially expressed between male control and MCT‐treated mice, both of which are pseudogenes: ENSMUSG00000074506 (Gm10705) and ENSMUSG00000094708 (Gm10359). To evaluate MCT‐induced changes across both sexes, expression data from male and female control mice and MCT‐treated mice were next pooled to look for the effects of MCT on skeletal muscle, independent of sex. In this analysis, 118 differentially expressed genes were identified, only 5 of which were upregulated in response to MCT: *Cyp26b1*, *Prickle2*, *Zfp800*, *Plscr4* and *Il‐16*. A volcano plot illustrates the split between downregulated and upregulated genes, and the complete list of differentially expressed genes are depicted in a heatmap (Figure 3A‐B). GSEA was used to identify important biological pathways and processes that may be responsive to MCT and identify the mechanisms of myofiber atrophy. A total of 26 pathways were identified (Figure 3C). The top 10 upregulated pathways are shown in Figure 3D and primarily include pathways associated with DNA damage and inflammation (e.g., HALLMARK_UV_RESPONSE_DN, HALLMARK_MITOTIC_SPINDLE and HALLMARK_INFLAMMATORY_RESPONSE), whereas pathways associated with oxidative phosphorylation, DNA repair and adipogenesis were downregulated (Figure 3E). These broader changes in pathways are reflected in the heatmap, which demonstrates a downregulation in genes related to mitochondrial function (e.g., mitochondrial ribosomal proteins *Mrpl42*, *Mrps24*, *Mrps21* and *Mrpl54*; subunits of the respiratory chain complex I *Ndufa3*, *Ndufc1*, *Ndufv3* and *Tmem126a*) and skeletal muscle (e.g., *Ckm*, *Mylpf*, *Myl4*, *Myl1*, *Myoz1* and *Ybx3*). Interestingly, some genes identified as significantly differentially expressed related to adipose tissue function, insulin sensitivity and metabolism, including *Zfp800* [26], *Dusp29* aka *Dusp27* aka *Dupd1* [27, 28], the adiponectin receptor (*Adipor1*) and *Aamdc*, a positive regulator of adipogenesis [29].

### 3.4 MCT Leads to Adipose Tissue Wasting in White Inguinal and Brown Interscapular Fat Stores in Males, Independent From Intestinal Distress or Reduced Food Intake

Although total body mass was significantly reduced in males, this was not explained by a corresponding loss in skeletal muscle mass of the TA or gastroc. Since cachexia also includes the loss of adipose tissue, we assessed the mass of three fat stores: inguinal (subcutaneous), gonadal (visceral) and interscapular brown fat. Sex and MCT treatment had significant main effects on the mass of all fat pads assessed. In males, the summed mass of all fat depots, plus the individual mass of inguinal fat and brown (interscapular) fat, was significantly reduced in MCT‐treated males compared with male controls, whereas there was no significant effect of MCT on female mice. In males, total fat depots were reduced by ~30%–40%. We next assessed whether changes in body and fat mass were due to off‐target toxicity of other organs such as the intestines, which are affected in cancer cachexia [30]. The lengths of the small and large intestines (measured with and without cecum) were measured in all treatment groups. No effects of MCT on intestinal length were found (data not shown). Next, the water content of stool was calculated to assess bowel wall oedema. In this experiment, 24‐h wet:dry stool measurements were taken at Weeks 2, 4, 6 and 8. No effects of MCT or sex on stool measurements were observed (data not shown).

**FIGURE 4:**
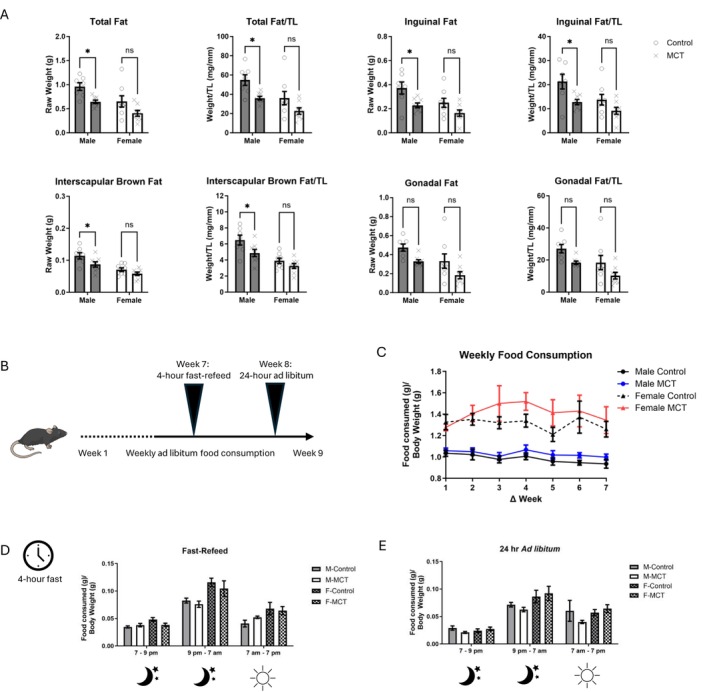
Physiological responses of adipose tissue to monocrotaline. (A) Terminal fat mass of the inguinal, gonadal and interscapular fat pads or summed weights (total fat). Both raw mass and mass normalized to tibia length (TL) are shown. (B) A cartoon depicting food intake experiments. (C) Weekly food consumption normalized to weekly body mass. (D) Fast‐Refeed measurements. The Fast‐Refeed study occurred during Week 7. Singly housed mice were deprived of food for 4 h prior to monitoring a 24‐h ad libitum feeding window at the described times. (E) Measurement of 24 h ad libitum feeding. The 24‐h ad libitum experiment took place during Week 8 in singly housed mice. Replicates for all feeding studies are *n* = 7–8 and are normalized to body mass. Data are shown as mean ± SEM. A two‐way ANOVA with Šídák's post hoc analysis (4A) or three‐way ANOVA (4C‐DE) was used to calculate statistical significance. *p < 0.05, **p < 0.01.

To evaluate feeding behaviour, we measured three feeding behaviours: weekly food intake, a 24‐h ad libitum measurement after a 4 h fast in Week 7 and a 24‐h ad libitum measurement with no fast in Week 8 (Figure 4B). When normalized to body weight, sex was a significant main effect (*p <* 0.0001) as females ate approximately 30%–40% more food per gram body weight than males; however, there was no effect of MCT on either sex (Figure 4C). To evaluate the drive to eat, a Fast‐Refeed experiment was completed during Week 7. Mice were placed on a 4 h fast, after which food intake was subsequently recorded within 3 windows: 7 pm–9 pm, 9 pm–7 am and 7 am–7 pm. As expected, mice ate more during the dark phase, and like the weekly feeding behaviours, females ate more than males (Figure 4D; main effects for time and sex, *p* < 0.0001). No effects of MCT on food intake were observed. Lastly, a 24‐h ad libitum measurement was conducted during Week 8. At this point, whole body mass is significantly lower in males. Similar trends to the Fast‐Refeed study were observed, with no significant effects of MCT on food intake, but main effects of time and sex (Figure 4E; *p* < 0.05). In summary, there were no significant effects from MCT on food consumption, stool output or intestinal length that could account for changes in body mass or fat stores.

### 3.5 Markers of Inflammation and Metabolic Reprogramming Are Upregulated in Male Mice in Response to MCT

**FIGURE 5:**
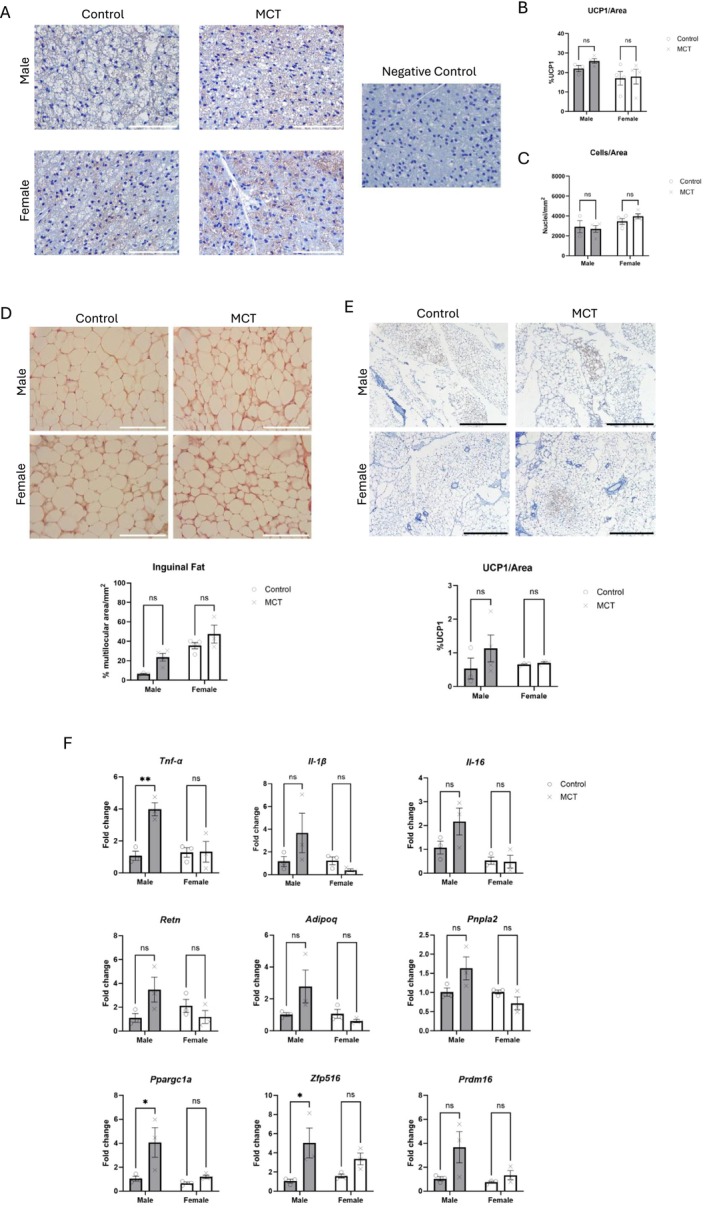
Analysis of metabolic dysfunction and inflammation in white and brown adipose tissue. (A) UCP1 staining of interscapular brown adipose tissue (BAT). Scale = 100 μm. A negative control lacking the primary antibody targeting UCP1 is included for each sex. (B) Quantification of nuclei/area in brown adipose tissue (*n* = 3–4). (C) Quantification of UCP1 signal within BAT (*n* = 3–4). (D) Representative images and quantification (bottom panel) of haematoxylin and eosin staining (H&E) of inguinal fat. Scale = 100 μm. The area of multilocular tissue was normalized to the total area measured to obtain a percentage of multilocular area/mm^2^ (*n* = 3–4). (E) Representative images and quantification of UCP1 staining of inguinal fat. Scale = 500 μm. (F) Expression of inflammatory and metabolic genes within the inguinal fat. Gene expression is shown as fold change relative to *Gapdh*. Data are shown as mean ± SEM. A two‐way ANOVA with Šídák's post hoc analysis was used to calculate statistical significance. *p < 0.05, **p < 0.01.

Inflammation and adipose tissue lipolysis and beiging (i.e., metabolic reprogramming of white adipose tissue to brown) have been linked to cancer cachexia [31]; therefore, we tested whether cardiac dysfunction via MCT also leads to adipose tissue inflammation, metabolic activation and/or beiging as a potential mechanism to explain the atrophy of adipose stores. We first examined BAT activity via staining for uncoupling protein 1 (UCP1). Based on immunohistochemical analysis, MCT did not have a significant effect on UCP1, though there was a main effect of sex (*p* = 0.0459; Figure 5A–B). Nuclei within a defined area were also measured as a proxy for cell size. Although MCT had no effect, there was again a main effect of sex on the number of nuclei/area in BAT (Figure 5C), suggesting that females have smaller adipocytes and/or more cells within BAT (*p =* 0.0006).

We next examined cellular changes of inguinal white adipose tissue (iWAT). Multilocularity was approximated via histology relative to total area. Although there was no significant effect of MCT on multilocularity within either males or females, main effects were determined for sex and MCT treatment (*p* < 0.05), suggesting that, as with the BAT, adipose tissue is significantly different between the sexes but also shows a treatment response (Figure 5D). We next stained for UCP1 within iWAT, but there were no significant differences in UCP1 levels (Figure 5E).

The expression of genes associated with inflammation, lipolysis, metabolism and browning was assessed in iWAT (Figure 5F). There was a significant interaction between sex and treatment on *Tnfα* expression, with 4× higher levels in MCT‐treated males. There was also a main effect of sex on *Il‐16* expression (*p* < 0.05). Significant interactions between sex and treatment were identified for *Retn* (codes for the adipokine, resistin) and *Pnpla2* (codes for adipose triglyceride lipase, Atgl). Main effects of MCT treatment on the expression of *Zfp516*, a transcription factor that drives transcription of *Ucp1* and is associated with tissue beiging [32], *Prdm16* (another regulator of *Ucp1* transcription) and *Ppargc1a* (codes for PGC1α) were observed. *Zfp516* and *Ppargc1a* were upregulated by 4–5 fold in MCT‐treated males. Together, these suggest that MCT induces inflammation and metabolic reprogramming of iWAT in males but not in females.

## 4 Discussion

Our study found that compared with males, female mice are protected from the effects of MCT on changes in total body weight and adipose tissue mass and inflammation; however, whole muscle mass, endurance and transcriptome data suggest weaker effects on skeletal muscle that were generally conserved between sexes. Our results are consistent with other studies that demonstrate cachexia does indeed cause a loss of fat mass in mice, and that adipose tissue loss occurs before lean mass [33, 34] and is likely due to sex‐dependent mechanisms. For example, in a mouse model of pancreatic cancer, female mice lost adipose tissue mass significantly later compared with male mice [35]. That cachexia manifests later in females is consistent with our findings using MCT.

Inflammation was induced in a sex‐dependent manner in the adipose tissue of male MCT‐treated mice. Sex‐dependent inflammation has been observed in other models of cachexia [35, 36], and thus, increased lipolysis in males via upregulated levels of inflammatory cytokines like TNFα could be a mechanism of fat loss in our model [37]. We also measured the expression of a novel cytokine that has recently been linked to cancer and sarcopenia, IL‐16. This cytokine binds to the CD4 receptor in immune cells and induces a chemotactic response. RNA‐seq of the gastrocnemius muscle identified *Il‐16* as one of the few upregulated genes in MCT‐treated mice (Figure 2E). It has recently been linked to sarcopenia and poor survival in gastric cancer patients [38, 39] and even obesity and adipogenesis [40]. IL‐16 could be an interesting therapeutic target in modulating inflammation and metabolism in cachexia.

The gross changes in brown and white inguinal fat depots suggest that males are more prone to MCT‐induced fat atrophy than females. This appears to be in part due to inflammation (i.e., *Tnfa*), but as *Zfp516* and *Ppargc1a*, two genes associated with metabolism and brown adipose tissue, were also upregulated, it is possible that MCT triggers a metabolic shift in white adipose tissue towards a catabolic state. Our UCP1 analysis showed no significant change in UCP1 levels in brown or white fat, though with three to four replicates, this analysis was underpowered. Based on the food intake measurements collected (Figure 4), changes in adipose tissue mass are not due to feeding behaviour, though indirect calorimetry and physical activity were not monitored.

One limitation of our study is that we did not measure the levels of the active metabolite, MCTP. Therefore, it is possible that males are metabolizing MCT to the toxic form more quickly than females; however, both sexes demonstrated signs of cardiovascular dysfunction (i.e., larger lung mass in both sexes and larger cardiomyocyte size in males), and both developed muscle atrophy (based on myofiber CSA). Though the RNA‐seq data did not yield any sex‐dependent changes in gene expression, it was interesting to see a general decline in muscle and mitochondrial‐related genes in the MCT‐treated mice, independent of sex. Reduced mitochondrial gene expression is particularly interesting as alterations in mitochondrial function and these mitochondrial changes manifest before muscle atrophy in ovarian cancer models [41].

Our study used a moderate dose of MCT at 200 mg/kg. This is a third of what other reports have used to induce cachexia in mice [13, 16]. At 200 mg/kg, there was no change in gross heart mass, which is observed at the higher concentrations [13]; however, lung mass was larger in both sexes, cardiomyocytes were larger in males, and a main effect of MCT was detected for expression of *Nppa*, a cardiac stress marker (Figure 1). These measures indicate subtle changes in the cardiovascular system at 200 mg/kg that are more pronounced at 600 mg/kg. The continued use of MCT as a model of hypertension and heart failure remains challenging, given the potential non‐specific effects that may be induced, a common concern among many pharmacological models of disease. Surgical models of heart failure to evaluate cardiac cachexia may be more appropriate to pursue.

Our study is the first to directly compare the sex‐dependent cachectic responses of mice to MCT and identifies key biological sex differences in this model of cardiac cachexia, particularly in skeletal muscle atrophy/weakness and fat loss. Although no differences were observed in gross skeletal muscle mass, the reduction in skeletal muscle CSA and overall changes in gene expression were generally consistent across sexes. More overt sex‐dependent responses were observed in adipose tissue, a strong contributor to the overall sex‐dependent loss in body mass seen in males. Metabolic rewiring in response to MCT as a model of hypertension/cardiac cachexia has not been evaluated before; however, given the sex‐dependent changes within adipose tissue in males, further examination of whether this represents a response to cardiac dysfunction or MCT itself should be addressed. Ultimately, these data demonstrate sex‐dependent responses to MCT, which should be considered in future studies.

## Ethics Statement

All animal procedures were approved by the Trent University Animal Care Committee.

## Conflicts of Interest

The authors declare no conflicts of interest.

## Supporting information


**Data S1:** Supporting information.
